# Update in Noninvasive Home Mechanical Ventilation: A Narrative Review of Indications, Outcomes, and Monitoring

**DOI:** 10.1155/2024/7013576

**Published:** 2024-07-03

**Authors:** Laura Tregidgo, Prasheena Naran, Eshrina Gosal, Rebecca F. D'Cruz

**Affiliations:** ^1^ Lane Fox Respiratory Unit Guys and St Thomas' NHS Foundation Trust, London, UK; ^2^ Department of Respiratory Medicine Barts Health NHS Foundation Trust, London, UK; ^3^ Department of Respiratory Medicine University College London Hospitals NHS Foundation Trust, London, UK; ^4^ Centre for Human and Applied Physiological Sciences King's College London, London, UK

## Abstract

Hypercapnic respiratory failure arises due to an imbalance in the load-capacity-drive relationship of the respiratory muscle pump, typically arising in patients with chronic obstructive pulmonary disease, obesity-related respiratory failure, and neuromuscular disease. Patients at risk of developing chronic respiratory failure and those with established disease should be referred to a specialist ventilation unit for evaluation and consideration of home noninvasive ventilation (NIV) initiation. Clinical trials demonstrate that, following careful patient selection, home NIV can improve a range of clinical, patient-reported, and physiological outcomes. This narrative review provides an overview of the pathophysiology of chronic respiratory failure, evidence-based applications of home NIV, and monitoring of patients established on home ventilation and describes technological advances in ventilation devices, interfaces, and monitoring to enhance comfort, promote long-term adherence, and optimise gas exchange.

## 1. Introduction

Noninvasive ventilation (NIV) delivered in the home environment is an effective treatment for patients with chronic hypercapnic respiratory failure. Clinical trials evaluating its application in a range of disease groups demonstrate that, with careful patient selection, it can improve clinical, patient-reported, and physiological outcomes. This narrative review provides an overview of the pathophysiology of respiratory failure, principles of noninvasive ventilation, applications of home noninvasive ventilation, and the evidence supporting the application of home mechanical ventilation (HMV) for patients with chronic obstructive pulmonary disease (COPD), obesity-related respiratory failure, and neuromuscular disease (NMD), and methods of monitoring patients established on HMV.

## 2. Principles of Ventilatory Homeostasis

The primary function of the respiratory system is to facilitate gas exchange by supplying oxygen and removing carbon dioxide (CO_2_) from the bloodstream to achieve ventilatory homeostasis. This process is achieved by two components: (1) the alveolar gas-exchange surface and (2) the respiratory muscle pump responsible for ventilation of the lungs [1]. The respiratory pump is comprised of inspiratory muscles, predominantly the diaphragm, and also intercostal and accessory muscles, airways, and lung parenchyma. Neural respiratory drive and ventilatory homeostasis are maintained by central respiratory control centres, which respond to feedback from central and peripheral chemoreceptors and mechano- and irritant-receptors in the chest wall and respiratory muscles, airway epithelium, and lung tissue [2].

**Figure 1:**
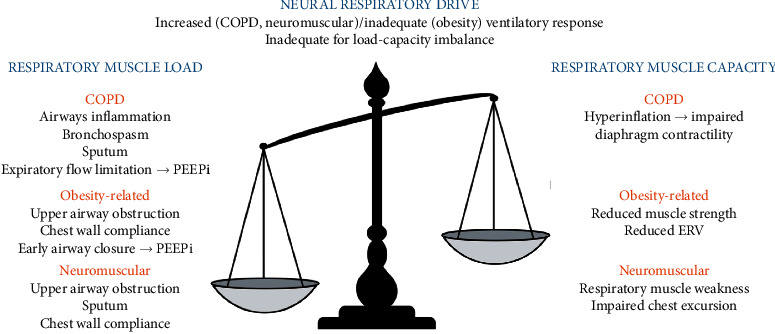
Schematic illustrating the pathophysiology of chronic respiratory failure caused by a load-capacity-drive imbalance of the respiratory muscle pump. COPD, chronic obstructive pulmonary disease; PEEPi, intrinsic positive end-expiratory pressure; ERV, expiratory reserve volume.

Hypoxaemic respiratory failure occurs when there is an impairment of gas exchange at the alveolar-capillary level, resulting in inadequate oxygenation. This can be explained by four mechanisms: alveolar hypoventilation, ventilation-perfusion mismatch, diffusion defects, or right-to-left shunt [1]. Hypercapnic respiratory failure is caused by an imbalance in the loads and capacity of the respiratory muscle pump and inadequate neural respiratory drive to maintain ventilatory homeostasis [3] (Figure 1).

Excess respiratory muscle loads can be classified into resistive, elastic, or threshold loads. Resistive loads occur in COPD, asthma, and cystic fibrosis as a consequence of airway inflammation, bronchospasm, and sputum production [4]. Resistive loads arise in obesity, NMD, and chest wall disease due to upper airway obstruction and reduced lung volumes and chest wall compliance [5]. Alveolar destruction in COPD imposes an elastic load and intrinsic positive end-expiratory pressure (PEEP_*i*_), caused by expiratory flow limitation in COPD and early airway closure in obesity, and creates a threshold load. Respiratory muscle capacity may be reduced due to hyperinflation and impaired force-generating capacity of the diaphragm, or intrinsic respiratory muscle weakness caused by NMD [6].

## 3. Principles of Noninvasive Ventilation

Chronic respiratory failure is managed with the optimisation of the underlying condition and with mechanical ventilation. The goal of the therapy is to achieve correction of gas exchange. This involves the delivery of positive airway pressure which may be achieved invasively, via endotracheal or tracheostomy tube, or using NIV, via the upper airway. Mechanical ventilation is used to control hypercapnia, and supplementary oxygen corrects hypoxaemia.

Inspiratory positive airway pressure (IPAP) is the peak airway pressure delivered during inspiration, and expiratory positive airway pressure (EPAP) is the pressure delivered during expiration and has the effects of maintaining airway patency and facilitating alveolar recruitment. In individuals with PEEP_i_, appropriately titrated EPAP can overcome this threshold load and improve the work of breathing, ventilator triggering, and patient-ventilator synchrony [7]. The difference between IPAP and EPAP is termed pressure support, which actively supports respiratory muscles and decreases work of breathing, overall acting to increase tidal volume and minute ventilation. This “bilevel” support contrasts with the fixed pressure delivered throughout the respiratory cycle by continuous positive airway pressure (CPAP), which is thus not a form of assisted ventilation. Inspiratory time (Ti) is set based on the underlying cause of respiratory failure, with a shorter Ti preferred for those with obstructive lung disease to permit adequate lung emptying and a longer Ti for those with NMD and obesity-related respiratory failure. A backup rate maintains ventilation when the patient does not initiate a breath, and consideration should be given to patient comfort as well as to optimising ventilatory control when this is set.

Different ventilator modes can be considered which can deliver fixed pressures (pressure-targeted ventilation) or a target tidal volume (volume-targeted pressure support). Pressure-targeted ventilation provides a preset IPAP and EPAP. Volume-targeted pressure support involves IPAP autotitration to achieve a preset target tidal volume, based on subjects' ideal body weight [8]. Gas exchange, patient tolerance, and sleep quality are comparable between pressure- and volume-targeted modes [8, 9]. Furthermore, volume-targeted pressure support can facilitate delivery of higher preset maximum IPAP without deleteriously impacting comfort [10]. Specialist ventilation units may consider a mode in which both IPAP and EPAP are autotitrated to abolish upper airway obstruction and control hypoventilation [11].

Consideration should be given to the interface to optimise comfort and minimise excess unintentional leaks. A wide range that covers the mouth, nose, or both are commercially available. Oronasal is the most commonly implemented interface amongst HMV users, and individual participant data meta-analyses indicate that there is no difference in gas exchange or adherence between oronasal and nasal [12].

## 4. Applications of Home Mechanical Ventilation

### 4.1. Chronic Obstructive Pulmonary Disease

#### 4.1.1. Pathophysiology of Respiratory Failure in COPD

COPD is a common respiratory condition and the third leading cause of death worldwide [13]. It is caused by inhalation of noxious particles, most commonly cigarette smoking and air pollution, which causes airway inflammation and alveolar destruction. The pathophysiological hallmark of COPD is expiratory flow limitation, which arises due to airway inflammation, bronchospasm, and mucus hypersecretion, which causes hyperinflation [14]. The development of chronic respiratory failure in COPD can be challenging to predict and is thought to affect approximately one-third of patients with severe disease [15]. Hypercapnic respiratory failure may present acutely *de novo* with respiratory acidosis or develop over time with compensated respiratory failure.

Chronic hypercapnia has both cardiovascular and musculoskeletal implications in COPD [16, 17] and is independently associated with mortality risk [18]. The aim of home NIV initiation is to correct hypercapnia and improve patient-centred outcomes, including hospitalisation risk, mortality, symptom burden, and health-related quality of life (HRQoL). Clinical practice guidelines on the use of domiciliary NIV to manage chronic respiratory failure in COPD are based on contemporary randomised clinical trials (RCT) which evaluated its application in patients with (1) stable hypercapnia and (2) persistent hypercapnia following acute hypercapnic episode requiring NIV, detailed as follows [19, 20].

#### 4.1.2. Stable Hypercapnic Respiratory Failure

Current international guidelines make a conditional recommendation for the use of domiciliary NIV in stable patients with COPD and chronic hypercapnia [21]. Early RCTs implementing low-pressure ventilation (average IPAP range: 12–14; EPAP range: 2–5) demonstrated no clear benefit on gas exchange, survival, hospitalisation, or exacerbation rate and could have a deleterious effect on HRQoL [22–24]. Kohnlein et al. then demonstrated that by targeting arterial CO_2_ reduction of at least 20% or <6.5 kPa (49 mmHg) using a high-intensity approach of higher pressures and backup rate (BUR) (mean IPAP 22; EPAP 5; BUR 16), nocturnal HMV improves daytime gas exchange occurring as early as 14 days after treatment initiation, compared to control-arm participants receiving optimised COPD care alone with no NIV [19]. Furthermore, those patients receiving HMV had lower all-cause 1-year mortality with no associated impairment of HRQoL. Further research to identify patient characteristics related to treatment success and adherence will continue to help improve patient selection for HMV in stable COPD.

#### 4.1.3. Persistent Hypercapnia following Acute Exacerbation

The benefits of HMV in patients requiring acute NIV for acute hypercapnic respiratory failure have been explored in two large RCTs. The RESCUE trial randomised inpatients admitted with life-threatening respiratory failure with persistent hypercapnia of >48 hours after liberation from mechanical ventilation to receive long-term oxygen therapy (LTOT) and NIV or LTOT alone [25]. In this study, gas exchange improved in those receiving HMV, however, there was no difference in time to readmission or death between the groups. It is possible that this was observed due to the inclusion of patients without chronic hypoxaemic respiratory failure and those in whom hypercapnia would naturally resolve during recovery from their acute illness and therefore had a better prognosis. Murphy et al. therefore designed the HOT-HMV to randomise patients with persistent hypercapnia 2–4 weeks following resolution of respiratory acidaemia to receive LTOT or LTOT and NIV [20]. This study demonstrated significant prolongation in time to readmission or death in the HMV + LTOT group compared to those receiving LTOT alone. In addition, the COPD exacerbation rate was lower in the HMV group. These trials highlight the importance of careful patient selection when considering HMV initiation for persistent postexacerbation hypercapnia.

Overall, there is robust evidence supporting the use of HMV in stable hypercapnic COPD patients and in those with persistent hypercapnia following an acute hypercapnic episode, which is reflected in international guideline recommendations [21]. Future research to investigate the optimal timing of NIV initiation may help to identify patients most likely to benefit from treatment. In addition, few trials have assessed NIV and its impact on long-term patient outcomes, which may provide further insight into patient selection.

### 4.2. Obesity-Related Respiratory Failure

Obesity-related respiratory failure (ORRF) is defined by the presence of obesity (BMI ≥30 kg/m^2^), sleep-disordered breathing (obstructive sleep apnoea (OSA) with an apnoea-hypopnea index of ≥5 events/hour, or nonobstructive hypoventilation with CO_2_ rise, or deep desaturation without obstruction), and daytime hypercapnia PaCO_2_ of ≥6 kPa (45 mmHg) in the absence of an alternative neuromuscular, mechanical, or metabolic explanation for hypoventilation [26]. It is common in the general population, with increasing prevalence and rising BMI, affecting >25% of those with a BMI of >40 kg/m^2^ [27]. Patients with ORRF may initially present with respiratory failure during an acute hospital admission, in whom long-term mortality is more than double that of individuals admitted with simple obesity [28]. A proportion is identified in the sleep and bariatric service outpatient setting [29].

#### 4.2.1. Pathophysiology of Obesity-Related Respiratory Failure

The pathophysiology of ORRF depends on changes to respiratory mechanics that arise as a result of fat deposition, and the subsequent impact this has on respiratory drive, alongside sleep-related changes noted in this cohort [30]. Work of breathing is impacted by the weight of the chest wall and surrounding abdomen, which is intensified in the supine position and thus during sleep [31]. Increased adipose deposition reduces lung compliance, diaphragmatic movement, and lung volumes (expiratory reserve volume and functional residual capacity) [30]. Early airway closure leads to gas trapping and PEEP_*i*_, which further increases the work of breathing and ventilation-perfusion mismatch [32]. Concurrent OSA arises due to adipose tissue deposition around the upper airway causing increased collapsibility, leading to obstruction during sleep [30]. In ORRF, the neural respiratory drive response to hypercapnia is blunted. This is hypothesised to be secondary to leptin resistance [33]. Leptin acts as a ventilatory stimulant, and as such, resistance results in decreased response to hypercapnia, which manifests as hypoventilation.

Sleep apnoea is more common in those with hypervolaemic states such as congestive cardiac failure and end-stage renal failure. This occurs due to chronic nocturnal hypoxaemia, which potentiates vascular damage, eventually leading to organ dysfunction and interstitial fluid retention [34]. When in supine position, fluid shifts rostrally to the thorax and neck as hydrostatic pressures within lower limb capillaries are reduced due to the reduced effect of gravity. This allows fluid to easily move from the extravascular (interstitial) to the intravascular compartment and is redistributed around the body [35]. In ORRF, where the respiratory drive is already diminished, the patient cannot compensate, resulting in further CO_2_ retention and progressive worsening of daytime hypercapnia [30].

#### 4.2.2. Ventilatory Management of Obesity-Related Respiratory Failure

Positive airway pressure therapy, alongside lifestyle modification (dietetic measures and weight management), is first-line treatments in patients with ORRF, with the overall goal of correcting sleep-disordered breathing, arterial hypoxaemia, and daytime hypercapnia [30, 36]. Weight management is the optimal treatment and can be curative, but this is often difficult to achieve independently [37]. Depending on local services, some patients may be considered for bariatric surgery, which is an effective medium-term weight loss strategy although weight gain can recur in subsequent years [38]. Furthermore, the limited evidence on the effect of bariatric surgery on sleep-disordered breathing indicates an ongoing need for positive airway pressure therapy postoperatively [39]. Both CPAP and NIV have been identified as effective treatment modalities for sleep-disordered breathing in obese subjects. Several factors should be considered when selecting which therapy to apply, including patient phenotype (simple OSA, hypoventilation with OSA, or lone hypoventilation), device cost, and device complexity.

In the short term (3 months), there appear to be no significant differences in physiological or patient-reported outcomes between CPAP and NIV in patients with ORRF, excluding those with persistent hypercapnia or significant oxygen desaturation despite CPAP. Piper et al. observed no differences in daytime PaCO_2_ reduction, weight loss, daytime somnolence, or treatment adherence between CPAP and NIV [40]. Using a primary outcome of treatment failure, as defined by nonadherence, hospital admission, or residual ventilatory failure (failure of PaCO2 to fall below 8 kPa (60 mmHg) or a rise of ≥1.3 kPa (10 mmHg), Howard et al. observed comparable improvements in daytime PaCO_2_, HRQoL, and treatment adherence [41]. Persistent ventilatory failure was seen in both cohorts, and this was related to the severity of ventilatory failure at presentation (measured by PaCO2 at initial presentation). Patients in the highest quartile (PaCO2 >8.3 kPa, 62 mmHg) had an eightfold increased risk of persistent ventilatory failure. In addition, patients with more severe OSA were less likely to have persistent ventilatory failure, supporting the hypothesis that correcting upper airway obstruction during sleep (using CPAP or NIV) results in treatment response. Notably, this study excluded patients with evidence of clinical hypoventilation (persistent hypoxaemia) and severe hypercapnia and participants' BMI was lower than in comparable trials [40, 42].

Medium- and long-term outcomes have been evaluated by Masa et al. in the Pickwick Project [42, 43]. This was a large, multicentre RCT comparing CPAP, NIV, and lifestyle measures in the treatment of ORRF with severe OSA (AHI >30 events/hour), initially using daytime hypercapnia as the primary outcome [42]. In the medium-term, both CPAP and NIV improved daytime hypercapnia in adherent (>4 hours usage/night) subjects compared to lifestyle measures alone. In the long term, NIV and CPAP were compared over a median follow-up of 5.4 years [43]. No differences were observed in the number of hospitalisation days per year (primary outcome), physiological outcomes (daytime PaCO_2_, PaO_2_, pH, or bicarbonate), weight loss, daytime somnolence, treatment adherence, incidence of cardiovascular events, or survival.

The Pickwick Project included only patients with severe OSA (AHI >30 events/hour), based on the hypothesis that those with more frequent obstructive nocturnal events would be more likely to respond to CPAP [42]. Therefore, the results for the efficacy of CPAP in this trial are not generalizable to all ORRF patients. Masa et al. have also evaluated ORRF patients without severe OSA (AHI<30 events/hour), comparing NIV to lifestyle modifications, and observed a significant reduction in daytime hypercapnia, serum bicarbonate, daytime somnolence, and HRQoL in those receiving NIV [44].

It is thus crucial to evaluate the patient's phenotype to guide the selection of positive airway pressure therapy. The aim of the therapy is to effectively control sleep-disordered breathing using an intervention that can be supported by local services and that the patient can adhere to. Patients with predominant OSA and normal or mildly elevated PaCO_2_ (<7 kPa, 53 mmHg) may be considered for CPAP, and those with hypoventilation and severe OSA and those with hypoventilation and mild or moderate OSA should be initiated on NIV as first-line therapy [45]. Consideration should also be given to device expense and availability, staff training and expertise, and setup facilities, with CPAP devices being less costly than NIV and can readily be setup in the outpatient department [45, 46].

### 4.3. Neuromuscular and Restrictive Thoracic Disease

#### 4.3.1. Pathophysiology of Respiratory Failure in Neuromuscular and Restrictive Thoracic Disease

Chronic respiratory failure develops in a range of hereditary and acquired neuromuscular disorders, which may be classified into slowly or rapidly progressive, due to respiratory muscle weakness. Understanding the rate of disease progression is important when determining the need for and timing of initiation of NIV. Duration of daily ventilator usage following HMV setup, rather than ventilation intensity, is key to optimising gas exchange in this cohort [47]. The most common rapidly progressive NMD is amyotrophic lateral sclerosis (ALS). Respiratory muscle involvement is typical and inevitably leads to respiratory failure, which is the leading cause of death [48]. Timely NIV initiation following the development of respiratory muscle weakness can improve quality of life and increase survival in patients without significant bulbar disease [49]. Patients with NMD may experience difficulty in effectively clearing respiratory secretions, which can lead to recurrent infection. Following evaluation of cough strength, for example, with peak cough flow [50], manual techniques including breath stacking and manually assisted cough and mechanical insufflation-exsufflation (“cough assist”) may be implemented [51]. Slowly progressive NMD includes postpolio syndrome, spinal muscular atrophy, myotonic dystrophy, and muscular dystrophies (including Duchenne muscular dystrophy and limb-girdle muscular dystrophy) [52]. Patients with these disorders are at variable risk of respiratory failure.

Restrictive thoracic disorders (RTDs) include pathologies which affect the architecture of the spine, sternum, or ribs. This includes congenital or acquired chest wall deformity and severe kyphoscoliosis. Ventilatory failure in RTD occurs due to decreased compliance of the chest wall, leading to a reduction in functional residual capacity. As a result, there is a compensatory increase in the work of breathing to achieve the same tidal volume, which can lead to hypoventilation.

#### 4.3.2. HMV in Neuromuscular Disease

Patients should ideally be referred to a ventilation unit prior to the development of symptoms or signs of respiratory muscle weakness to enable close monitoring and timely initiation of HMV. Symptoms of respiratory muscle weakness include breathlessness, orthopnea, recurrent respiratory infection, sleep disturbance with unrefreshing sleep, daytime somnolence and fatigue, and persistent morning headache. Respiratory muscle surveillance can be performed by measuring vital capacity (VC), sniff nasal inspiratory pressure (SNIP), maximal inspiratory and expiratory pressures (MIP and MEP), peak cough flow (PCF), and overnight oximetry or capnography to detect nocturnal hypoventilation (Table 1) [50, 53, 54]. The aims of NIV in this cohort are to improve survival, symptoms, and HRQoL [55].

It is challenging to perform prospective clinical trials by recruiting patients with rare and/or rapidly progressive disease; therefore, data on optimal timing of HMV setup and associated prospective data on short-, medium-, and long-term outcomes are limited. Current clinical practice is therefore largely guided by case series, observational studies, and consensus expert opinion. Current guidelines recommend that NIV should be initiated in patients with NMD presenting with symptoms of sleep-disordered breathing and either daytime hypercapnia (PaCO_2_ >6 kPa, 45 mmHg), respiratory muscle weakness, or evidence of sleep-disordered breathing on overnight oximetry [57]. This practice is supported by data from Ward et al., who aimed to evaluate whether nocturnal hypercapnia in the presence of daytime normocapnia was a valid indication for NIV [58]. Subjects included those with congenital NMD and chest wall disease with a VC of less than 50% predicted, or symptoms suggestive of nocturnal hypoventilation. Subjects were daytime eucapnic and overnight transcutaneous CO2 monitoring confirmed nocturnal hypercapnia (peak transcutaneous CO_2_ > 6.5 kPa, 49 mmHg). At 12 months, 70% of patients in the control group developed ventilatory failure requiring NIV.

The landmark trial of home NIV in ALS was undertaken by Bourke et al.. Subjects with symptomatic daytime hypercapnia or orthopnea with MIP of <60% predicted were randomised to receive usual care or additional home NIV [49]. Symptoms, HRQoL, and respiratory muscle tests were monitored for up to 3 years. In the NIV group, quality of life remained above 75% baseline for the majority of the study, with improvements mainly related to sleep quality. Survival benefits were observed in the NIV group compared to standard care amongst those with normal or moderate bulbar dysfunction (median survival 216 days in the NIV group compared to 11 days in the control group). Whilst this survival benefit was not conferred in those with severe bulbar dysfunction, comparable improvements in HRQoL were suggested potential benefits of NIV in this cohort despite continual physical decline.

It has also been shown that NIV confers survival and HRQoL advantages amongst patients with other types of NMD. A small observational study undertaken by Simonds et al. evaluated the survival benefit of NIV in patients with Duchenne muscular dystrophy (DMD) [59]. Subjects were established on HMV based on symptomatic/proven daytime hypercapnia (PaCO_2_ >7 kPa, 53 mmHg) and confirmed nocturnal hypercapnia. Of the subjects established on NIV, 73% remained alive at 5 years. Despite functional limitations, HRQoL was comparable to that of age-matched male controls with regard to mental health and social factors.

Based on a range of evidence sources, including observational, RCT, and consensus expert guidance, patients with NMD should be referred for assessment at a specialist ventilation unit early in their disease trajectory to evaluate their risk of developing respiratory failure. Patients with symptoms and objective evidence of respiratory muscle weakness and/or sleep-disordered breathing should be established on HMV, and in certain cohorts, this may improve survival and HRQoL.

#### 4.3.3. HMV in Restrictive Thoracic Disease

Respiratory failure is the leading cause of death in patients with RTD [60]. Initiation of HMV in this cohort is largely guided by clinical expertise and small clinical trials' datasets. Owing to the relatively small patient cohort, the evidence for NIV is largely a combination of retrospective and prospective observational and uncontrolled clinical trials, which indicate that home NIV improves physiological and clinical outcomes, including gas exchange, HRQoL, and risk of acute hospitalisation [61, 62].

### 4.4. Location of HMV Setup

Conventionally, HMV is initiated during an inpatient hospital admission. As increasing numbers of patients are being established on HMV following recent clinical trials and increasing numbers of regional ventilation units and expertise, outpatient or home setups have been evaluated to explore clinically- and cost-effective alternatives to inpatient initiation.

In patients with COPD, Duiverman et al. conducted an RCT which demonstrated noninferiority of home versus hospital initiation for patients with stable hypercapnic respiratory failure [63]. This trial showed comparable exacerbation and hospitalisation rates between home and hospital setups, as well as a significant reduction in cost associated with home setup. This trial points towards home initiation as a noninferior, cost-effective option for NIV initiation in the COPD cohort.

The recent OPIP trial explored the safety and cost-effectiveness of outpatient HMV setup in stable patients with ORRF [64]. Murphy et al. identified no significant difference in medium-term cost-effectiveness between outpatient and inpatient setup, and comparable PaCO_2_ control between both the groups. Whilst long-term outcomes remain under investigation, current evidence indicates that HMV setup should be guided by local team resources. Irrespective of setup location, all patients should be reviewed on a regular basis to monitor adherence, disease control, and interface fit (particularly relevant in the context of intentional weight loss) and offer ongoing lifestyle modifications, including dietetic support and referral to bariatric surgical services where appropriate and available.

Patients with NMD may present with advanced disease with marked disability, and as such may prefer to minimise time spent in hospital to reduce the associated emotional burden and risk of nosocomial infection [65]. Hazenberg et al. evaluated home NIV initiation in patients with NMD or chest wall deformity and identified noninferiority of a home compared to in-hospital setup regarding PaCO_2_ reduction and HRQoL, as well as significant cost saving in favour of home setup.

### 4.5. Monitoring HMV Users

#### 4.5.1. Clinical Evaluation

Patients established on home NIV are monitored on an outpatient basis at a specialist ventilation unit, typically every 3–6 months depending on local protocols. Some services may offer telephone or video consultations in combination with home visits for patients with advanced disease, for whom transport may be challenging. Enquiry is made into the aforementioned symptoms of chronic respiratory failure and sleep-disordered breathing, unscheduled healthcare utilization, including treatment of respiratory infections or exacerbation of airways' disease, and hospital admissions.

#### 4.5.2. Software Analysis and Telemonitoring

Most newer devices can be setup to continuously record a wide range of technical parameters and store data in a cloud-based system to enable remote monitoring. Older devices store data on SD cards, which can be downloaded at outpatient appointments [66]. These data can supplement patients' descriptions of adherence and interface fit, as well as optimise ventilator settings using data on tidal volume for pressure-targeted ventilation and mean and 90^th^ percentile delivered pressures for volume-targeted ventilation. Disease control for patients with sleep-disordered breathing can be evaluated using the residual AHI. These data can thus be used as a form of telemonitoring, which yields potential opportunities to improve compliance and reduce hospitalisations and exacerbations [67, 68], and may represent cost-saving opportunities for health services [69]. These data may also be used to predict respiratory deterioration, as highlighted by Blouet et al. who observed an increased respiratory rate in the days preceding an exacerbation in HMV users with COPD [70].

#### 4.5.3. NIV Troubleshooting

Patients may report noisy air leaks, eye dryness or infection, or pressure-related skin damage, which must prompt an interface review. Physical examination for erythema or pressure sores should routinely be undertaken and encouraged by patients at home. Mask cushions or pressure relieving dressings and warning against overtightening straps can help reduce the risk of developing pressure sores [71]. A range of interface designs and sizes are available and should be selected to suit patients' face shape and comfort. A nasal or minimal contact interface can be used in instances where patients are claustrophobic [72]. A humidifier may relieve discomfort caused by upper airway dryness. A ramp, whereby pressures are increased over a preset time, can be added for comfort. Accurate assessment of leak is also important for device optimisation; significant leak can result in inaccurate tidal volume estimation and patient-ventilator asynchrony, ultimately impacting on gas exchange and patient tolerance [73]. Inpatient review with overnight evaluation may be offered if troubleshooting cannot rectify issues in the outpatient setting.

#### 4.5.4. Patient-Reported Outcomes

Breathlessness is common in patients with chronic respiratory failure and can be distressing and disabling [74]. Patient-reported outcome (PRO) tools can help with symptom reporting, and these may be general or disease-specific (Table 2) [75]. Psychosocial aspects including the effects of treatment burden especially in patients with multimorbidity can impact adherence [76]. Patients who are younger, have a mental health diagnosis, and are from socioeconomically deprived backgrounds may need additional support to promote adequate HMV compliance [77].

#### 4.5.5. Physiological Measurements

PaCO_2_ measurement is required to diagnose chronic respiratory failure. For patients who are established on NIV, physiological response should be regularly monitored. This may be achieved using arterial, capillary, or transcutaneous sampling. Transcutaneous monitoring involves a sensor warmed to a temperature of 40–44°C to cause local arterialisation, which when applied with an algorithm, provides measures of CO_2_, SpO_2_, and heart rate. Transcutaneous CO_2_ (TcCO_2_) values have been shown to correspond with PaCO_2_ within a clinically acceptable range [86]. Whilst it should not be used to diagnose respiratory failure, TcCO_2_ has a valuable role in monitoring following HMV setup, can be performed in the outpatient and home settings, and can limit the need for painful arterial sampling [63].

Home overnight oximetry measurements can be used to evaluate mean SpO_2_ and time-spent SpO_2_ below 90% (*T*<90%), with acceptable thresholds based on individuals' pathophysiology, and the SpO_2_ trace is used to identify rapid deep desaturations (indicative of upper airway obstruction) or periods of prolonged desaturation (indicative of hypoventilation) [87]. Persistently abnormal findings despite HMV adherence should prompt a clinical review and reevaluation of device settings and interface.

The aforementioned tests of respiratory muscle strength are used to diagnose and monitor patients with neuromuscular disorders (Table 1). Measurement of PCF is valuable in the assessment of expiratory muscle strength and effective secretion clearance; a reduced value may predispose to chest infections and increased mortality risk [88]. Values of >250–270 L/min have been proposed as a threshold for risk of recurrent chest infections and for consideration of a daily secretion management schedule including manual techniques and mechanical insufflation/exsufflation [89].

#### 4.5.6. Physiological Techniques under Evaluation

Patients with chronic respiratory failure are at high risk of respiratory infection, hospitalisation, and death. Advanced physiological markers that can predict respiratory deterioration could facilitate timely clinical review and treatment initiation, and thus prevent hospitalisation. Emerging techniques are as follows:

1. Parasternal electromyography (EMG_para_): Neural respiratory drive (NRD) cannot be measured directly in living subjects; therefore, surrogate measures are required. Respiratory muscle electromyography can be used, with both invasive and surface techniques applied in patients with airways' disease and obesity [90, 91]. During severe COPD exacerbations, EMG_para_ is closely associated with patient-reported and physician-defined clinical deterioration and can be used to predict COPD patients who are safe for discharge and at lower risk of early hospital readmission [92, 93]. Overall, EMG_para_ is a feasible and acceptable home-based technique, which tracks patient-reported and physiological recovery from severe COPD exacerbation [94].
2. Forced oscillation technique (FOT): It involves the application of small-amplitude sonic oscillations during tidal breathing via a mouthpiece to quantify respiratory system reactance and resistance [95]. Application in the home setting has shown it to be an acceptable measurement for COPD patients, with good daily adherence, as observed by Walker et al. [96]. It may also be used to identify expiratory flow limitation in COPD and optimise HMV pressure to abolish it by using a novel auto-titrating ventilatory mode [7]. The clinical utility of FOT in optimising gas exchange, predicting respiratory events, and reducing risk of hospitalisation remains under investigation.
3. Physical activity monitoring: It is a simple and noninvasive technique that can be achieved by using commercially available or advanced accelerometer devices typically worn on the nondominant wrist. Such devices can quantify physical movement and actigraphy indices can be used to evaluate sleep quality in the home setting. Physical activity monitoring can identify changes during periods of acute illness and has been used extensively in clinical trials of COPD patients [97–99]. Further data are needed to evaluate the long-term effect of HMV on functional limitation in patients with chronic respiratory failure.

## 5. Conclusions

Hypercapnic respiratory failure is a complication of commonly encountered diseases and arises due to an imbalance in the loads and capacity of the respiratory muscle pump. Clinicians should be aware of indications to refer patients to a specialist ventilation centre for assessment and consideration of home noninvasive ventilation, considering risk factors, symptoms, signs, and physiological evaluation. Robust clinical trials demonstrate that home NIV can improve clinical, patient-reported, and physiological outcomes in selected patients with COPD, obesity-related respiratory failure, and neuromuscular disease. Technological advances have seen developments in novel ventilator modes, interfaces to optimise comfort, and remote monitoring of detailed technical parameters, all of which serve to support clinicians in delivering personalised, patient-centred care.

## Figures and Tables

**Table 1:** Objective assessment of respiratory muscle weakness [50, 53, 54, 56].

Indicator of respiratory muscle weakness	
Peripheral oxygen saturation	SpO_2_ <94% *OR* SpO_2_ <92% (if known lung disease) *OR* subjective symptoms (especially orthopnea) If any present, urgent evaluation with arterial blood gas required
Arterial blood gas	PaCO_2_ >6 kPa (45 mmHg), urgent referral to ventilation centre PaCO_2_ ≤6 kPa (45 mmHg) with subjective symptoms (especially orthopnea), refer to ventilation centre for evaluation with sleep study and respiratory muscle testing

Respiratory muscle weakness indicating the need for NIV initiation	

FVC or VC	FVC or VC <50% predicted *OR* FVC or VC <80% predicted with subjective symptoms (especially orthopnea) and a fall of >10% between seated and supine
SNIP	SNIP <40 cm H_2_O *OR* SNIP <65 cm H_2_O in males, or <55 cm H_2_O in females plus subjective symptoms (especially orthopnoea) *OR* decrease by >10 cm H_2_O over 3 months
PCF	PCF <160 L/min

FVC, forced vital capacity; PaCO_2_, arterial partial pressure of carbon dioxide; PCF, peak cough flow; SNIP, sniff nasal inspiratory pressure; SpO_2_, peripheral oxygen saturation; VC, vital capacity.

**Table 2:** Examples of available generic and disease-specific questionnaires.

Questionnaire	Type	Description
Modified Borg Scale [78]	Generic	Quantifies breathlessness intensity at the current point in time. Scored from 0 to 10
Medical Research Council (MRC) Dyspnoea Scale [79]	Generic	Categorises functional limitation. Grades 1 to 5
Severe Respiratory Insufficiency Questionnaire (SRI) [80]	Disease-specific: Respiratory failure	49 items covering respiratory complaints, physical functioning, attendant symptoms and sleep, social relationships, anxiety, psychological well-being, and social functioning. Scored from 1 to 5 for each item
The Maugeri Foundation Respiratory Failure Questionnaire (MF-28) [81]	Disease-specific: Respiratory failure	28-item questionnaire assessing 3 domains: Daily activities, cognitive function, and invalidity Scores 1 for positive responses
S^3^-NIV Questionnaire	Disease-specific: Respiratory failure	11 items assessing symptoms related to chronic respiratory failure and HMV use. Scored from 0 to 4 on each item with the average score multiplied by 2.5
COPD Assessment Test (CAT) [82]	Disease-specific: COPD	8 items covering symptoms, sleep disturbance, and disease impact on daily activities. Scored from 0 to 5
Individualised Neuromuscular Quality of Life Questionnaire (INQoL) [83]	Disease-specific: NMD	45 items covering muscle disease symptoms, impact on areas of life, and positive and negative effects of treatment
Sleep Ap**ne**a Quality of Life Index (SAQLI) [84]	Disease-specific: Sleep-disordered breathing	35 items assessing daily functioning, social interactions, emotional functioning, and symptoms Scaled scoring system
Epworth Sleep Score (ESS) [85]	Disease-specific: Sleep-disordered breathing	8 items requiring participants to rate the likelihood of sleeping during activity. Scored from 0 to 24

COPD, chronic obstructive pulmonary disease; HMV, home mechanical ventilation; NMD, neuromuscular disease.
